# Psychedelic training program development: lessons from the evolution of the EMBARK program

**DOI:** 10.3389/fpsyg.2025.1637887

**Published:** 2025-09-03

**Authors:** William Brennan, Melissa Field, Catherine Schon, Amir Inamdar, Alex Kelman

**Affiliations:** ^1^Consultant, New York, NY, United States; ^2^Consultant, Overland Park, KS, United States; ^3^Cybin, Inc., Toronto, ON, Canada

**Keywords:** psychedelic therapy, psychedelic medicine, psychedelic treatments, training program development, evidence-based training approaches, EMBARK model of psychedelic treatment

## Abstract

The field of psychedelic medicine is currently developing evidence-based standards and core competencies for training providers who offer psychedelic treatments. This article contributes to this effort by describing the iterative development of the EMBARK training program. This program has been developed by Cybin, Inc. for training session monitors in clinical trials of psychedelic treatments. The EMBARK training program has been used in two successfully completed clinical trials and three ongoing trials. This article details each of the three phases of the EMBARK training program’s development, including their core components, what worked well, lessons learned, and the rationale for any changes made. Innovations included in EMBARK^CT^, the most recent iteration of the training used in Cybin’s Phase 3 trials, are also discussed. These innovations include the use of artificial intelligence (AI) with expert human oversight to identify the need for additional support and training for session monitors. Finally, recommendations and implications for the field are discussed in this article.

## 1 Introduction

As psychedelic treatments progress toward regulatory review and potential approval, the question of how to effectively train psychedelic practitioners has received increased attention (Argento et al., 2024; Barber et al., 2024; Brennan and Belser, 2024; Dames et al., 2024a, 2024b; McGuire et al., 2024; Mocanu et al., 2022; Poppe et al., 2025; Rosenbaum et al., 2024; Villiger, 2024; Wilson-Poe et al., 2024). There is a widely recognized need to develop training programs that will meet the anticipated post-approval demand for trained psychedelic practitioners (Knepler et al., 2024).

A key part of addressing this need is the development of training standards, core practitioner competencies, and certification requirements that support safe and efficacious psychedelic care. In the United States and Europe, several groups have begun this process by soliciting perspectives from recognized experts in the field (e.g., Aicher and Gasser, 2024; BrainFutures, 2024; Gardner and Noble, 2024; Jacobs et al., 2024; McGuire et al., 2024; Passie et al., 2025; Perez Rosal et al., 2024; Phelps, 2017). Their work to date has laid important groundwork for creating the agreed-upon training standards for the field.

In addition to considering expert perspectives, the standards and competencies used in the field should have a basis in empirical evidence. One way to facilitate this is by evaluating the training approaches for psychedelic session monitors that have been found to support safety and efficacy in clinical trials. To support this evaluation, the field would benefit from greater transparency about the approaches to training used in successful trials (Brennan et al., 2023).

The current article serves that unmet need by examining the iterative development of the EMBARK psychedelic session monitor training program, which has been used in several successful clinical trials conducted by Cybin, a neuropsychiatric drug development company, and others (Back et al., 2024). This article will present various elements of the EMBARK training program, describe how they have evolved across successive iterations of the training program, and provide the rationale for their evolution. It also describes the challenges encountered, the solutions implemented, and the lessons learned from them. By offering in-depth insights into a program used to train session monitors across several successful trials, this article will provide valuable data that support the development of evidence-based standards for the field.

## 2 Overview of the EMBARK model

The EMBARK training program is based on the EMBARK model of psychedelic treatment. EMBARK is a trans-drug, transdiagnostic model of psychological support designed for use during psychedelic treatments. It was first developed for Cybin-sponsored clinical trials (Brennan and Belser, 2024; Brennan and Belser, 2022). The model has served as the basis for several trial-specific adaptations of EMBARK that apply a particular drug to a specific clinical indication. For example, the EMBARK approach has been applied to the treatment of major depressive disorder (MDD) using psilocybin-like molecules such as Cybin’s CYB003, a proprietary deuterated psilocin molecule (Brennan and Belser, 2024).

The core elements of the EMBARK model that are present in all adaptations include six clinical domains that comprise its name (Existential-spiritual, Mindfulness, Body aware, Affective-cognitive, Relational, and Keeping momentum) and its four ethical care cornerstones (Trauma-informed care, Culturally competent care, Ethically rigorous care, and Collective care). The six clinical domains represent the wide variety of potentially beneficial treatment experiences a participant may have when given a psychedelic drug in a supportive setting. EMBARK’s multidomain structure recognizes that each participant will have a unique treatment experience and prepares session monitors to conceptualize and support them in a flexible and responsive way. EMBARK’s four ethical care cornerstones reflect the model’s foundational commitment to ethical practice. Guidelines for providers on how to align their practice with each cornerstone are woven into all aspects of each EMBARK adaptation for a specific clinical indication.

Another key feature of EMBARK is that it has been designed to facilitate the incorporation of clinical interventions and established mechanisms of change from non-psychedelic evidence-based treatments (EBTs) and empirically supported treatments (ESTs). Each EMBARK adaptation for a specific clinical indication instructs session monitors to apply concepts from EBTs and ESTs that are most relevant to the content that arises in each participant’s unique treatment experience. This tailoring process is organized by the model’s six clinical domains. For example, a depressed participant having a treatment experience that falls within the “affective-cognitive” domain may be best supported by a cognitive behavioral or emotion focused therapy-based approach, while another having an experience more germane to the “mindfulness” domain may benefit more from an acceptance and commitment therapy-based approach.

To provide this type of participant-centered care, the EMBARK training program has aimed to provide session monitors with thoughtful, high-quality training. Its goal has been the same as that of the EMBARK model itself: to prepare session monitors to work ethically and efficaciously with the broad spectrum of emergent phenomena that may arise in psychedelic treatment.

## 3 Overview of the EMBARK training program

This article will discuss the evolution of the EMBARK training program by describing three phases of its development:

- The *original EMBARK training* was designed for small groups of session monitors with no prior psychedelic therapy experience.
- The *EMBARK crossover training* for session monitors who had previously worked on other psychedelic trials or who had completed other psychedelic training programs.
- EMBARK for Clinical Trials (*EMBARK^CT^*) was designed to be scalable for multisite Phase 3 trials conducted across several continents in multiple languages.

For each phase, this article will describe the components of the training, discuss what worked well, detail the lessons learned, and provide the rationale for any changes made from a previous phase. The perspective provided therein was developed through discussions among EMBARK team members and outside collaborators who worked on each phase, as well as through other inputs as noted.

## 4 Original EMBARK training

The first iteration of the EMBARK training was developed to support two trials that began in late 2022 and early 2023. One was an investigator-initiated trial led by Back et al. (2024) at the University of Washington, assessing the efficacy of psilocybin in burnout and depression related to coronavirus disease 2019 (COVID-19) in frontline healthcare workers. The second was a Cybin-sponsored Phase 2b trial assessing the safety and efficacy of CYB003 in the treatment of major depressive disorder (MDD).

The Cybin trial reported strongly positive topline outcome data, with 79% of participants who received two 12-mg doses of CYB003 in remission from MDD at 6-week follow-up, compared to 20% of the control group (*n* = 32) with full results currently being written up for publication (see Krempien et al., 2024). Dr. Back’s trial (Back et al., 2024) also reported positive outcomes, with a between-group mean difference in Montgomery–Åsberg Depression Rating Scale (MADRS) score reductions of −12.00 (*n* = 30; *p* < 0.001).

All session monitors enrolling in the training cohort for the Cybin trial (CYB003-001) were required to undergo an interview with a Cybin EMBARK core faculty member to ensure they had the appropriate experience and fit. Out of 15 qualified applicants, 1 applicant was denied entry into the program due to a lack of transferable skills. The accepted cohort included 14 licensed or license-eligible psychotherapists, a psychiatrist, and a psychiatric nurse practitioner. The cohort trained for the University of Washington trial (Back et al., 2024) included 11 practitioners with diverse professional backgrounds (psychotherapists, medical doctors, physician assistants, and nurse practitioners). No session monitor on either trial had previously worked on a psychedelic clinical trial.

The EMBARK team at Cybin, in collaboration with Dr. Back, developed the structure of the original EMBARK training. It required trainees to complete the following components:

- Self-guided review of the trial-specific EMBARK treatment manual, which included specifications on molecule and indication
- Asynchronous review of 12 video modules, each created by a different subject matter expert, on topics including MDD, working within each of EMBARK’s six clinical domains, and practicing in alignment with each of EMBARK’s four ethical care cornerstones
- Live Q&A sessions with these subject matter experts after watching each video
- Completion of a 3-day in-person training
- This included study molecule and protocol training with a daylong group experiential session in Holotropic Breathwork. During the experiential session, trainees were paired in groups of two. They had a chance to both provide support and be supported in a non-drug-induced non-ordinary state experience that approximates those elicited by classic psychedelic drugs—something that has been suggested as a helpful component of session monitor training (Villiger, 2024).

In addition to the above training, Cybin also provided approximately 8 h of asynchronous clinical trial and International Council for Harmonization-Good Clinical Practice (ICH-GCP) training for each session monitor in efforts to bolster their readiness for clinical trial compliance. A trainee who completed all the aforementioned training components was considered pre-certified as an EMBARK session monitor. Once a pre-certified session monitor began work on a Cybin trial, they were required to do the following during study conduct:

- Complete three educational consultation sessions (either as a dyad or an individual session monitor) with an expert consultant during their work with their first trial participant
- Attend monthly peer consultation meetings as a means to support each session monitor’s learning and development. In these meetings, one session monitor team presented a participant case while the other session monitors asked thoughtful questions, provided suggestions and feedback, and inquired about ways they could provide support to the presenting session monitor team.

**Figure 1:**
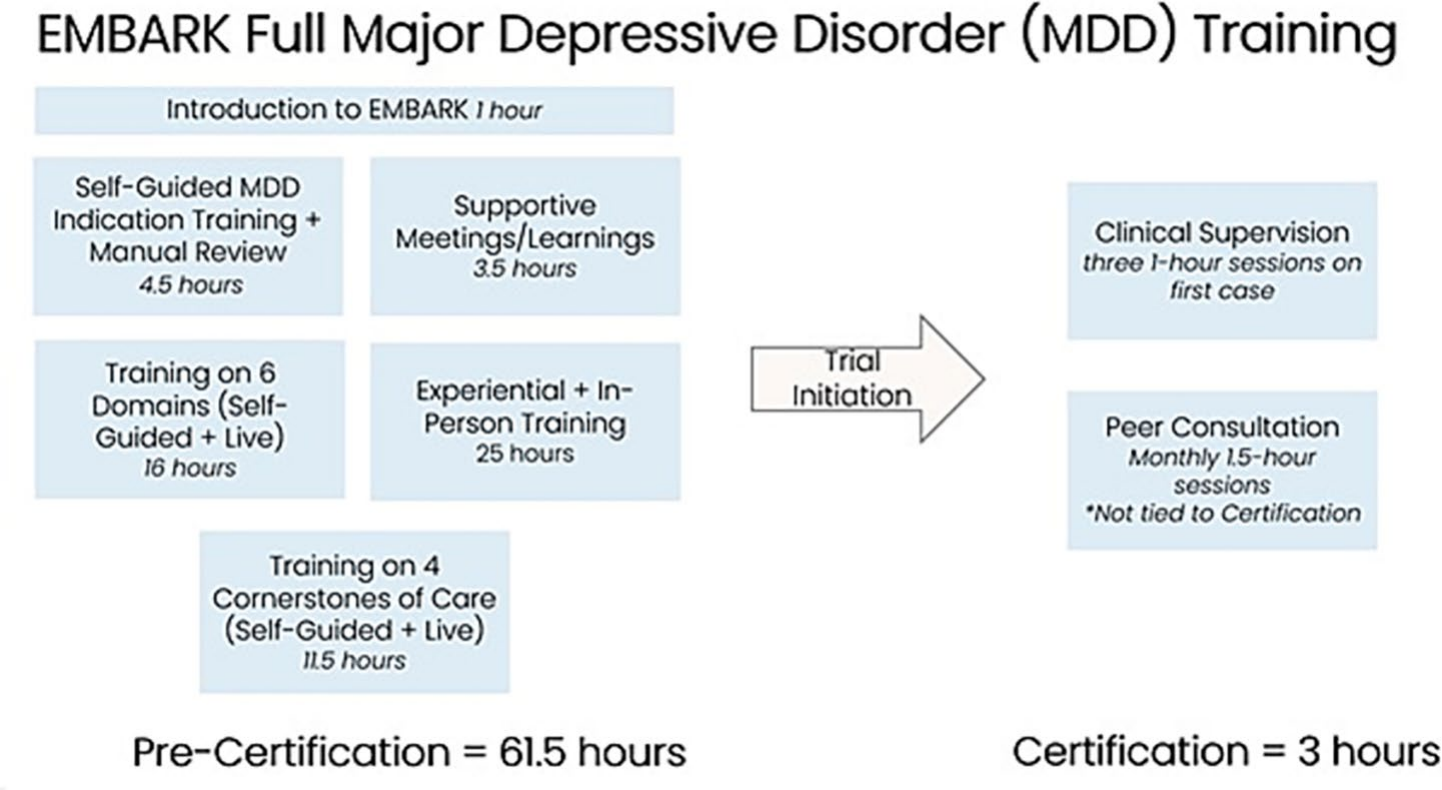
Full major depressive disorder (MDD) training.

Completing the three educational consultation sessions conferred full EMBARK certification onto a session monitor, which was considered a necessary credential to continue working on any current or future Cybin trials (see Figure 1).

### 4.1 Original training: what worked well

Elements of the first approach to EMBARK training that worked well were determined through soliciting iterative feedback from trained session monitors and continual reflection by program staff. They included:

- *Faculty with diverse expertise*. Rather than relying on one or a few trainers to deliver training content, training content delivered by multiple faculty members provided session monitors with an opportunity to learn in a manner that approximated a university seminar course or other comprehensive learning contexts.
- The video content created by these faculty members was later made freely available on www.embarkapproach.com as *EMBARK Open Access*, an informational training course on psychedelic treatments.
- *Early cohort cohesion building in an in-person training.* Session monitors benefited from the opportunity to come together in person early in their training and build relationships that would become the foundation for mutual support and collaborative learning throughout the trial.
- *Experiential training*. The group breathwork experience provided session monitors with a more direct, first-person understanding of the unique needs a participant might have while in a non-ordinary state. It also fostered a deeper sense of interpersonal connectedness among session monitors, which strengthened mutual support throughout the trial.
- *Clinical Supervision/Educational consultation.* In a follow-up survey, session monitors reported that both the mandated and as-needed consultation sessions were an invaluable support in making clinical judgment calls on challenging cases.
- *Peer consultation*. In the same follow-up survey, session monitors also reported that meeting regularly with their peers to discuss their work with participants provided a helpful source of support.
- *Iterative feedback from session monitors throughout the trial.* Session monitors provided continual feedback through formal evaluation forms submitted after each live session and by relating challenges to educational consultants, who then brought these concerns to leadership.

### 4.2 Original training: lessons learned

The first EMBARK training approach also had limitations that were improved upon in later iterations. These included:

- *Budgeting and scalability.* Training elements, such as in-person group trainings and mandated educational consultation for all new session monitors, were too resource-intensive for Phase 3 trials being conducted at many sites across several countries.
- *Unnecessary session monitor burden.* Some of the basic content covered in the EMBARK training modules would likely prove to be duplicative to session monitors with previous training and experience on psychedelic trials by other sponsors.
- *Inadequately integrated training content.* Although learning from a variety of expert faculty presented benefits, it also made the training content feel somewhat fragmented for some session monitors who wanted the training to give them a more integrated sense of their role. This feedback often contrasted the fragmentation of the training approach with the more well-defined session monitor role delineated in the session monitor manual.

The EMBARK crossover training and EMBARK^CT^ training were developed as responses to these limitations. The specific improvements made to each training will be discussed in the following sections.

## 5 EMBARK crossover training

A crossover training approach was developed to provide a more focused EMBARK training to session monitors with prior training from a well-established psychedelic facilitation training program by other organizations. Therefore, the crossover training assumes that trainees have a foundational understanding of the role of a psychedelic session monitor and requires that they hold a Certificate of Completion from a reputable psychedelic training program. This abbreviated approach succeeded in reducing duplicative foundational content, thereby alleviating the training burden placed on session monitors. It also succeeded in reducing the total cost of training incurred by the sponsor for ensuring that session monitors had competencies that they had already developed in other training programs.

The EMBARK crossover training was first used to train seven session monitors at a second research site that was brought on midway through Cybin’s aforementioned CYB003-MDD Phase 2b trial. This new group of session monitors consisted of six licensed or license-eligible psychotherapists and one licensed psychiatrist. See Table 1 for details on what was retained and excluded from the original EMBARK training.

**Table 1:** Elements of the EMBARK crossover training.

Retained from original EMBARK training	Excluded
Required interview with an EMBARK core faculty member Self-guided review of treatment manual Asynchronous review of 12 video modules by subject matter experts (carried over from the original training) Monthly peer consultation group Three mandated educational consultation sessions As-needed educational consultation	Live Question and Answer (Q&A) sessions with subject matter experts In-person training (molecule/protocol training provided to session monitors at the remote site initiation visit)
Prerequisites from outside training	Novel elements
Certificate of completion from a psychedelic therapist/session monitor training program First-person experiential training in a legal, supported modality involving a non-ordinary state of consciousness (waived for trainees with prior clinical trial experience)	Two synchronous, online 2.5-h “advanced practice” training sessions with core EMBARK faculty that included Q&A on video module content with an emphasis on its practical application An additional video on how to use the EMBARK treatment manual for this trial, created in response to previous session monitors’ questions about how to apply the training content in an integrated way

**Figure 2:**
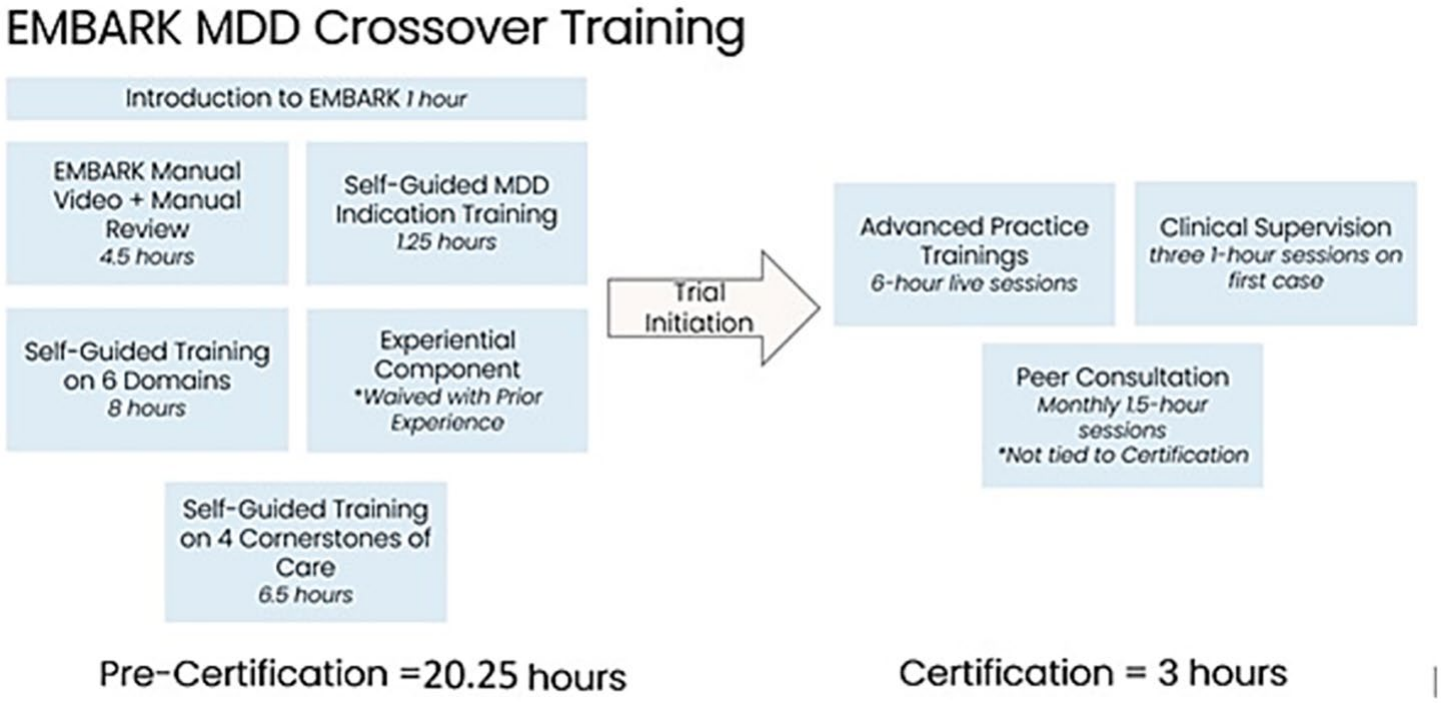
EMBARK MDD crossover training.

After the CYB003 Phase 2 trial concluded, the session monitors who had undergone this crossover training were brought back to work on another Cybin trial, which is currently ongoing as of the publication of this manuscript. This trial examines the safety and efficacy of CYB004, Cybin’s dimethyltryptamine (DMT) analog, as a possible treatment for generalized anxiety disorder. The additional training required for this trial consisted of two more 2.5-h advanced practice sessions. One session reviewed the EMBARK treatment manual written for this trial, and the other brought in session monitors who worked with another DMT-like molecule in an earlier trial (conducted by Small Pharma) to present on the participant experiences they observed. Educational consultation has not been mandated for the CYB004 Phase 2 trial, though it is still available upon request (see Figure 2).

### 5.1 Crossover training: what worked well

The EMBARK crossover training demonstrated the following advantages over the original EMBARK training:

- *Improved budgeting and scalability.* The in-person training components that were replaced with synchronous, remote training sessions were less resource-intensive for the sponsor.
- *Reduced session monitor training burden.* Session monitors with previous training on psychedelic treatments benefited from reduced training requirements and a reduction of duplicative content.
- *Increased focus on practical skill-building.* While the original training’s live Q&A sessions focused on understanding the video content, the crossover training’s advanced practice sessions focused more on grasping the specifications of what session monitors were asked to do on this trial. It used vignettes and roleplays to develop skills, rather than engaging in more conceptual discussions about aspects of psychedelic treatments.
- *Greater emphasis on the treatment manual.* Shifting the focus of the live sessions toward practical skill-building and away from the broad content of the videos also contributed to an increased focus on the specific manualized approach being used in the trial. This helped session monitors develop a clearer sense of their role, which likely improved clinician self-efficacy and treatment fidelity.

### 5.2 Crossover training: lessons learned

- *Failure to more actively integrate session monitors’ prior experience and training.* The crossover training could have made a greater effort to understand session monitors’ existing skill sets and understanding of their role at the start of the trial. This would have allowed core faculty to tailor the advanced practice sessions to cover the material that would be most supportive in training session monitors to work within the EMBARK approach.
- *Potential lack of camaraderie building.* The crossover training was used with a group of session monitors wo had existing relationships carried over from working together at their site on other trials and thus had an established group rapport. However, had this not been the case, the abbreviated design of this training, with its lack of in-person events, may have failed to build a rapport of mutual support and co-learning in a newly formed session monitor group.
- *Challenges of requiring first-person experiential training.* The process of assessing incoming session monitors’ prior experiential training prompted internal conversations about this requirement. We considered the impact of continuing this requirement into Phase 3 trials, which would span a broader geographical range and include cultures and jurisdictions with varied attitudes toward personal engagement with non-ordinary states. We also considered that not all trainees would be medically cleared to participate in experiential training. We removed this requirement to allow for greater accessibility for the Phase 3 trial EMBARK training.

## 6 EMBARK for clinical trials (EMBARK^CT^)

An evolution of EMBARK training was created in preparation for Cybin’s Phase 3 trials with its CYB003 molecule. EMBARK^CT^ (for “clinical trials”) was designed to be a further streamlined training that could be deployed to train session monitors for simultaneous trials at numerous sites across several countries. It was also continually honed so that it could hew closely to advice that the U.S. Food and Drug Administration (FDA) had recently issued for psychedelic research, both in the draft guidance it issued in June 2023 and in its July 2024 advisory committee’s reflections on Lykos’ new drug application for 4-methylenedioxymethamphetamine (MDMA) treatment of PTSD (U.S. Food and Drug Administration, 2023; Wilkinson and Sanacora, 2025).

All previous iterations of the EMBARK training program helped to form the development of the EMBARK^CT^ scaled session monitor training effort for Cybin-sponsored Phase 3 pivotal trials. The EMBARK^CT^ training program retained many of the key components that worked well, while modifying and improving other aspects based on lessons learned.

We ensured that the following elements from prior iterations were retained in EMBARK^CT^:

- Applicant interviews;
- Content drawn from the diverse subject matter expertise provided by the original EMBARK faculty;
- Educational consultation and peer consultation;
- Improved budgeting and scalability;
- Reduced session monitor training burden;
- Well-integrated training content that emphasizes practical skill-building;
- Consistent integration with training manual content.

**Figure 3:**
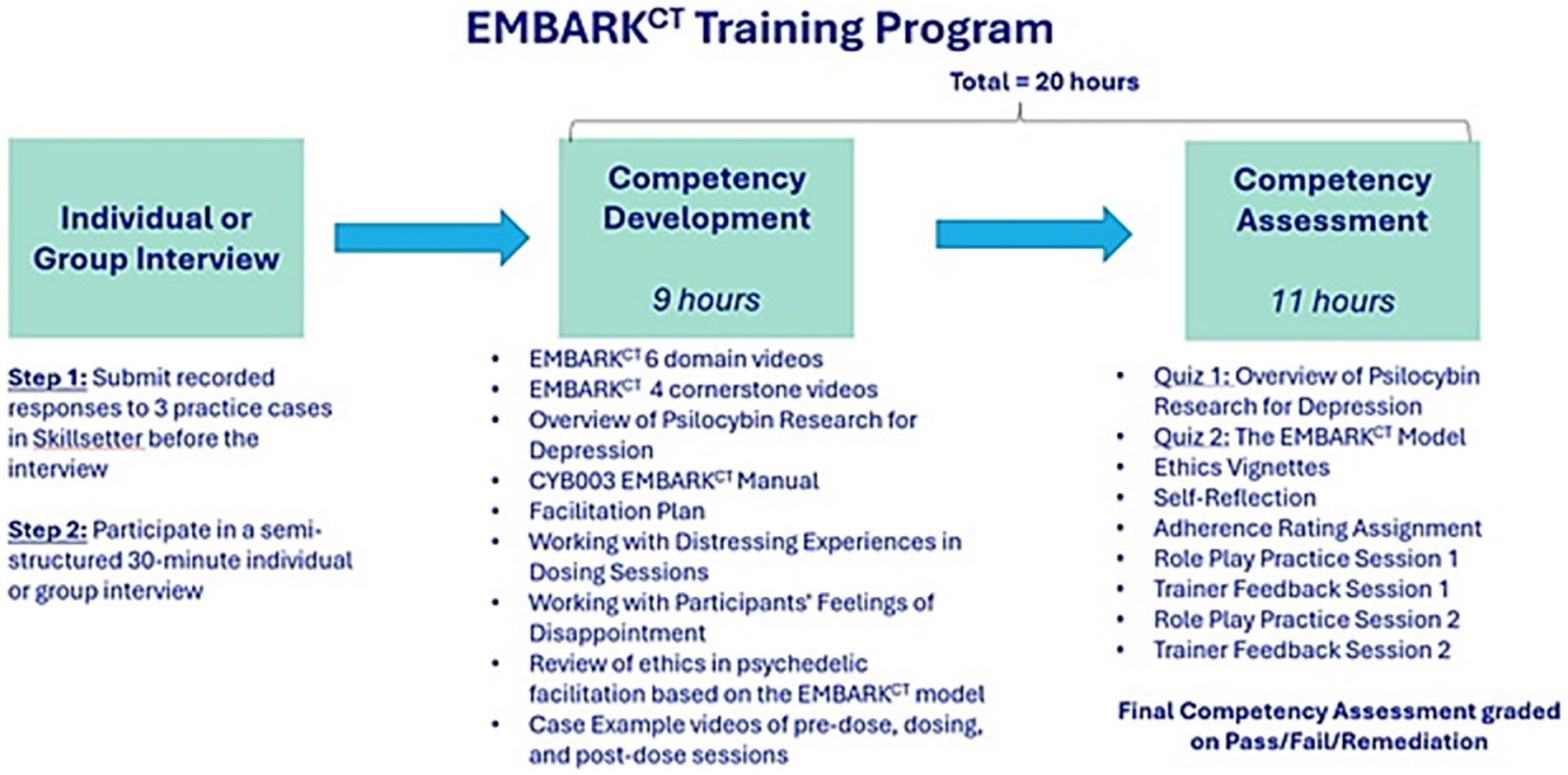
EMBARK^CT^ training program.

**Figure 4:**
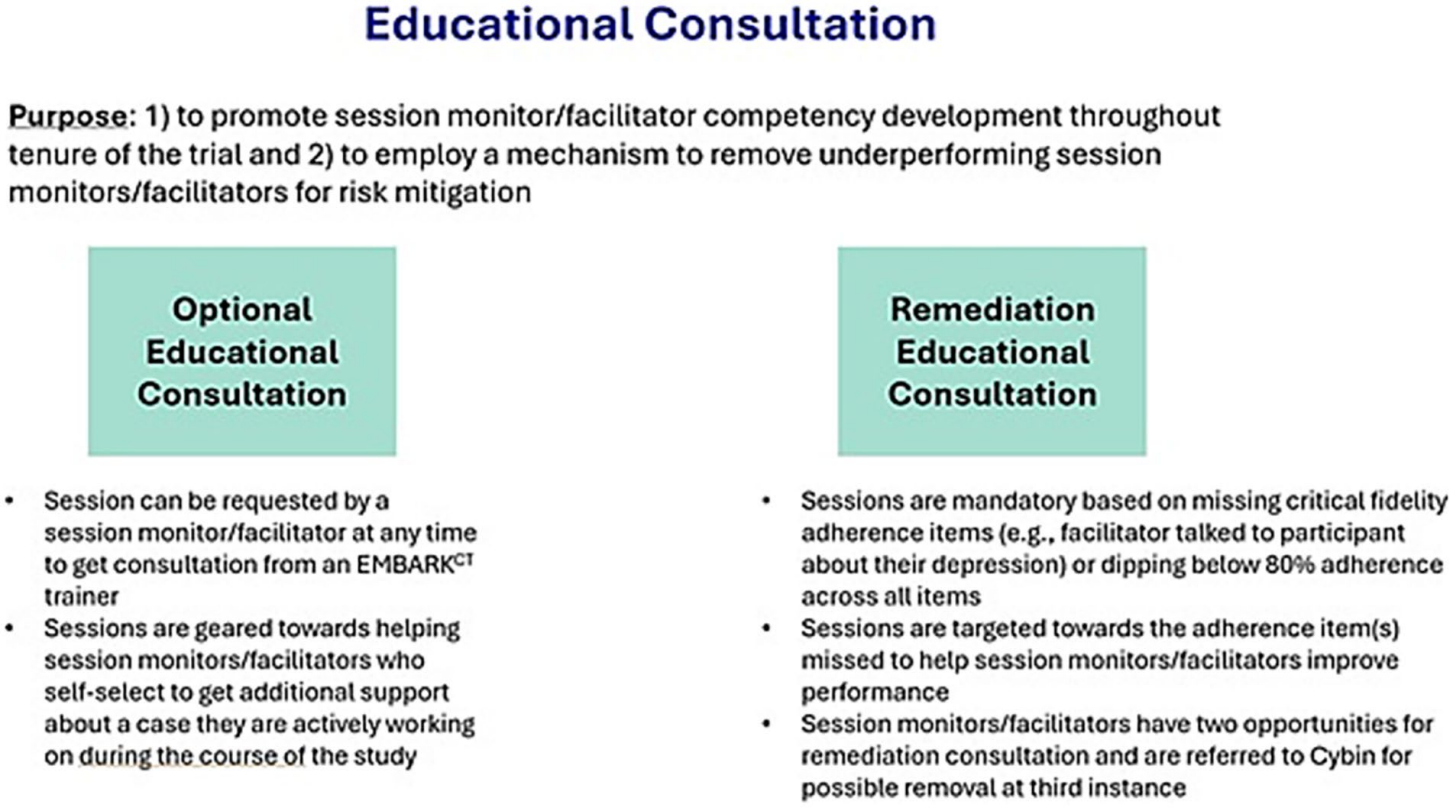
Educational consultation.

Training in EMBARK^CT^ differs from previous iterations of EMBARK training across the following areas:

- *Review of applicants’ recorded responses to session vignettes prior to interview.* Prior to their first meeting with an EMBARK^CT^ trainer, each applicant views video recordings of treatment events that may be encountered in a clinical trial. They then record a brief response to each video, capturing how they would act as a session monitor in that moment. Their interviewer then reviews their responses prior to the interview, allowing for another layer of assessment of the applicant’s skill set.
- *Requirement of prior foundational psychedelic training for applicants.* We require that all applicants complete an approved foundational psychedelic treatment training with a duration of at least 6 h from another training program prior to beginning the EMBARK^CT^ training. This allows us to focus the EMBARK^CT^ training on the specifications of the EMBARK approach used on the CYB003-MDD trial, rather than beginning with the basics. Any session monitor without this foundational training is offered enrollment in a 6-h Fluence training course ^1^ at sponsor expense or another program of their choosing.
- *Streamlined session monitor manual*. The EMBARK team at Cybin has collaborated with Fluence to develop an updated session monitor manual that presents the treatment approach from the Phase 2 trials in a more focused and accessible way, while making minor adjustments in the approach in response to FDA guidance issued in the interim (Brennan et al., 2024). Session monitor fidelity adherence checklists were also made available to assist them in adhering to the treatment approach.
- *Updated Learning Management System (LMS) training content*
- Content from both the session monitor manual and the original EMBARK video modules has been woven together into more concise videos that cover the EMBARK domains and care cornerstones.
- Session monitors are required to watch additional Fluence video content on the basics of psilocybin treatment for depressive disorders and review the CYB003 specifications within the manual.
- Session monitors are asked to reflect on and respond in writing to clinical vignettes that raise questions about ethical practice, in addition to a set of questions about how they have navigated ethical issues previously in their clinical practice.
- Session monitors are provided with the fidelity adherence criteria used on the trial, and they are asked to apply these criteria to a series of case demonstration videos as a way of familiarizing themselves with what will be expected of them on the trial.
- *Additional roleplay training*
- As part of their training, session monitors are paired and required to meet twice to conduct and debrief a total of six 15-min roleplays of different session types conducted by each monitor: pre-dose, dosing, and post-dose. These meetings are recorded and analyzed by Fluence’s AI, which provides feedback on each session monitor’s performance that is discussed in a live session with a trainer.
- The final component of training is a competency assessment in which session monitors meet in a live session with a trainer to complete a final 20-min roleplay. This roleplay is evaluated in real time by a trainer using the study fidelity adherence criteria. The trainer then provides feedback to the session monitor and decides whether to certify them.
- *Addition of a streamlined training track for experienced session monitors*. A streamlined version of the EMBARK^CT^ program is available to candidates with substantial prior experience and strong demonstrated comprehension of the session monitor role. This assessment is made during the candidate’s initial interview. The streamlined track makes the recorded partner roleplays and trainer feedback session optional, easing scheduling demands for qualified candidates. To ensure readiness, candidates on this track undergo a more extensive competency assessment at the end of the streamlined training (see Figures 3, 4).

### 6.1 EMBARK^CT^ : incorporation of AI to signal needs for monitor remediation training

Another defining feature of EMBARK^CT^ is the use of AI, not just for safety monitoring and fidelity adherence monitoring, but also to detect the need to either remove a session monitor with a substantiated allegation of misconduct or to provide remediation and educational consultation to session monitors whose work is insufficiently adherent to the treatment approach. All treatment sessions (pre-dose, dosing, and post-dose sessions) are analyzed shortly after they occur by mpathic’s AI-based system, which was trained on the EMBARK treatment approach used in Cybin’s Phase 3 CYB003-MDD trials with data collected from their Phase 2 trial. This system, which involves expert human oversight alongside AI review, offers scalable, unbiased, consistent, and near real-time monitoring of treatment fidelity in the provision of psychological support, effectively closing a gap identified in previous psychedelic trials by the FDA.

Additionally, real-time feedback on adherence facilitates the identification of session monitors who may benefit from additional training or support in their role. Remediation educational consultation is required when either of the following occurs:

- A session monitor’s adherence rating falls below 80% in any phase of treatment (pre-dose, dosing, or post-dose sessions).
- A session monitor misses an adherence item deemed critical by study staff (e.g., discussing the possible effects of the study drug).

**Figure 5:**
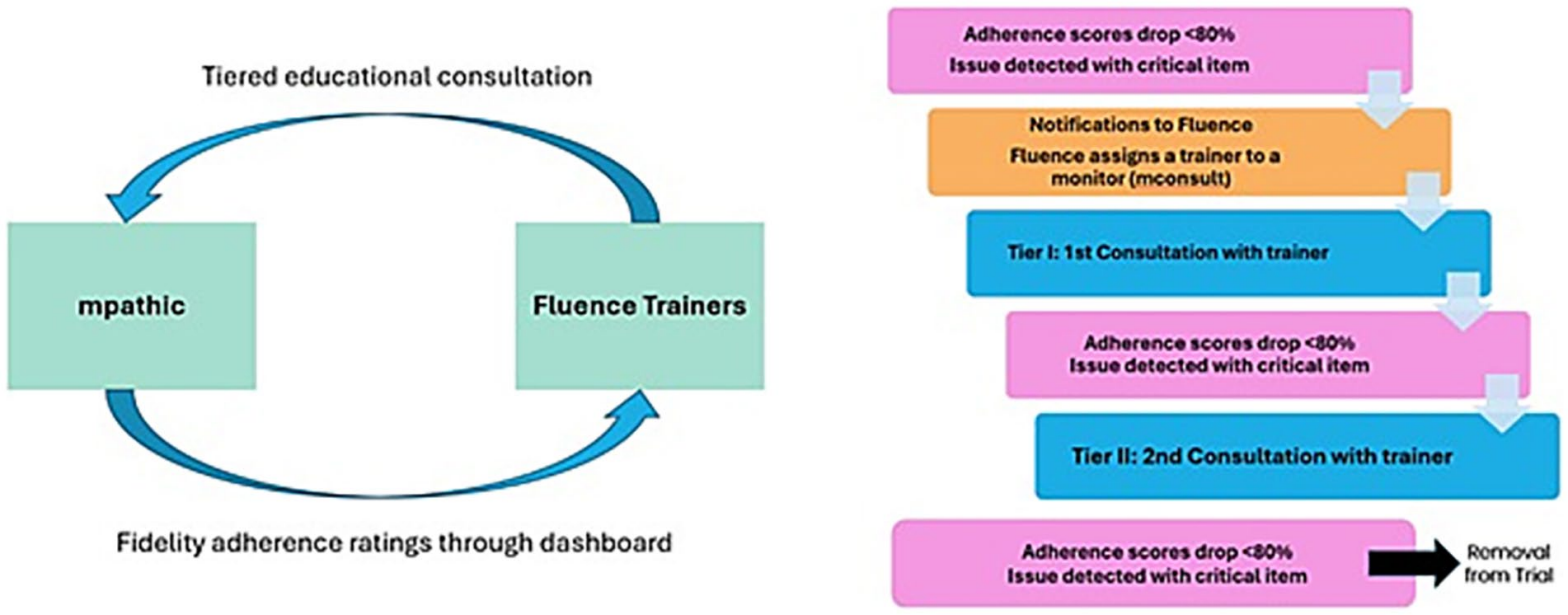
EMBARK^CT^ adherence and safety monitoring and intervention.

If a session monitor is found to be non-adherent three times, they are considered for possible removal from the trial. If a session monitor is detected speaking or acting in a way that is harmful to a participant or poses a safety risk, the issue is escalated. This is confirmed with the site PI, and the session monitor is removed from the trial and their EMBARK certification revoked. Additional actions may be taken by the site and/or regulatory or licensing authorities (see Figure 5).

### 6.2 EMBARK^CT^ : educational and peer consultation processes

All session monitors are encouraged to request educational consultation with an EMBARK^CT^ trainer at any time during study conduct if they have any questions about their work with a participant or about their role generally.

Session monitors also receive *required* educational consultation when they fail to meet key adherence criteria or drop below 80% in overall treatment adherence. These consultation sessions are structured in a manner that targets the specific treatment component that requires remediation. The session monitor reviews targeted self-study materials on a range of important skills for session monitors to complete prior to meeting with an educational consultant.

Additionally, monthly peer consultation group meetings are incorporated during the study conduct for each research site. Session monitors are encouraged to participate in these meetings as a means to support one another’s learning and development. As in previous iterations of the EMBARK training, these meetings consist of session monitors from each trial site meeting to discuss clinical questions that have arisen in their work with participants.

## 7 Implications for the field

The following section discusses several key lessons from the development of the various iterations of the EMBARK training program. They are offered in service of the efforts to develop approaches to psychedelic practitioner training that build upon training sessions that have supported treatment efficacy and participant safety in practice. Still, when considering these lessons, it is important to keep in mind that they are simply reflections of those involved in the development of EMBARK training programs and have not been formally tested as hypotheses about what contributes to an effective training program. Additionally, the EMBARK^CT^ program has only recently been deployed and has yet to undergo a completed trial.

*Offering post-training educational consultation.* EMBARK-trained session monitors have consistently expressed that the opportunity to meet with an educational consultant during their work on a trial was pivotal to their success in their role. Our team thus prioritized finding a way to continue to offer it as we scaled up to Phase 3 trials, which has so far proven possible with the integration of the AI-based referral system discussed earlier, alongside the opportunity to seek optional educational consultation at any time during trial conduct.

*Favoring practical education over didactics.* EMBARK training has shifted its center away from video-centric learning with content-based Q&A sessions toward roleplays and trainer feedback sessions. This change has given session monitors more opportunity to practice specific skills before using them with participants. It has also provided trainers with more insight into the specific training and support needs of each individual session monitor, allowing for personalized attention.

*Enhanced session monitor screening through a rigorous interview and competency assessment process during training.* Bringing the right people into training is perhaps the most important factor in predicting its outcomes. EMBARK^CT^’s multistep onboarding process allows for more engagement with the session monitor and their capacities before they are brought onto the trial.

*Setting the right tone when building relationships with session monitors.* We have viewed all components of our trainings—from in-person retreats to online roleplay sessions—as a kind of parallel process that models how session monitors are expected to engage with participants. The feedback we have received indicates that session monitors perceived this intention and that it positively impacted their feelings about working on Cybin trials. This emphasis on building relationships with session monitors has also opened the door to session monitors working on multiple Cybin trials, deepening their expertise in EMBARK with each one, with some even becoming trainers and educational consultants within the program.

*Synergizing session monitor training with other innovations used in trials.* Although the primary reason for the inclusion of mpathic’s AI system was to assess fidelity adherence to the EMBARK model, its secondary function as a referral mechanism for educational consultation demonstrates the value of integrating other trial supports to improve ongoing session monitor training and oversight.

## 8 Limitations

One important limitation to note is that the EMBARK training has been designed specifically for use in the context of clinical trials, where the goals and constraints differ from those of real-world clinical practice. Training for competence in a trial setting differs from preparing practitioners for independent, long-term clinical work with patients. Caution should be used when applying the findings in this article outside of the controlled conditions of a clinical trial. In a trial context, participant well-being is supported by intensive monitoring, including the AI-based system described in this article. At the same time, this protective function may be served by more extensive provider training in real-world settings.

Also, since this training program has not been developed for post-approval settings, its design should not be used as a model for reducing costs in those settings. While cost and scalability are essential topics for the field, those questions fall outside the scope of the current project and require separate, thoughtful engagement (Marseille et al., 2022; McCrone et al., 2023).

## 9 Conclusion

As the field of psychedelic treatments develops competencies and standards for the training of practitioners, it would be well-served by learning from the training approaches used in completed trials with efficacious outcomes. The present article offers information on the three phases of the EMBARK training program, as well as perspectives from those involved in their development, for consideration and analysis. In doing so, it aims to create evidence-based psychedelic practitioner training approaches that support the thoughtful and responsible evolution of these promising new treatments.

## Data Availability

The original contributions presented in the study are included in the article/supplementary material, further inquiries can be directed to the corresponding author.

## Footnotes

^1^ The name of the Fluence training is "Introduction to Psychedelic Therapy" and can be found at this URL if more information is required: https://www.fluencetraining.com/content/2892737-1.
